# A conserved pattern in plant‐mediated interactions between herbivores

**DOI:** 10.1002/ece3.1922

**Published:** 2016-01-21

**Authors:** Jing Lu, Christelle A. M. Robert, Yonggen Lou, Matthias Erb

**Affiliations:** ^1^ Root Herbivore Interactions GroupDepartment of BiochemistryMax Planck Institute for Chemical EcologyHans‐Knöll‐Str. 2107745JenaGermany; ^2^ Institute of Insect SciencesZhejiang UniversityZijingang Campus, Yuhangtang Road 866Hangzhou310058China; ^3^ Institute of Plant SciencesUniversity of BernAltenbergrain 213013BernSwitzerland

**Keywords:** Genetic variation, herbivory, indirect interactions, induced defense, plant resistance

## Abstract

Plant‐mediated interactions between herbivores are important determinants of community structure and plant performance in natural and agricultural systems. Current research suggests that the outcome of the interactions is determined by herbivore and plant identity, which may result in stochastic patterns that impede adaptive evolution and agricultural exploitation. However, few studies have systemically investigated specificity versus general patterns in a given plant system by varying the identity of all involved players. We investigated the influence of herbivore identity and plant genotype on the interaction between leaf‐chewing and root‐feeding herbivores in maize using a partial factorial design. We assessed the influence of leaf induction by oral secretions of six different chewing herbivores on the response of nine different maize genotypes and three different root feeders. Contrary to our expectations, we found a highly conserved pattern across all three dimensions of specificity: The majority of leaf herbivores elicited a negative behavioral response from the different root feeders in the large majority of tested plant genotypes. No facilitation was observed in any of the treatment combinations. However, the oral secretions of one leaf feeder and the responses of two maize genotypes did not elicit a response from a root‐feeding herbivore. Together, these results suggest that plant‐mediated interactions in the investigated system follow a general pattern, but that a degree of specificity is nevertheless present. Our study shows that within a given plant species, plant‐mediated interactions between herbivores of the same feeding guild can be stable. This stability opens up the possibility of adaptations by associated organisms and suggests that plant‐mediated interactions may contribute more strongly to evolutionary dynamics in terrestrial (agro)ecosystems than previously assumed.

## Introduction

Plants respond to herbivore attack by increasing the synthesis of defensive metabolites and proteins, reconfiguring their primary metabolism and adjusting their growth patterns (Howe and Jander 2008; Zhu‐Salzman et al. 2008; Machado et al. 2013). Many of these responses extend from the site of attack to nonattacked tissues and persist even after the attack is over (Heil and Ton 2008; Gómez et al. 2010). By consequence, herbivore attack can influence other plant‐associated organisms, including other herbivores. Plant‐mediated interactions between herbivores are increasingly recognized as important determinants of herbivore community composition and multitrophic interactions (Rodriguez‐Saona et al. 2005; Kaplan and Denno 2007; Poelman et al. 2008; de Rijk et al. 2013).

The outcome of plant‐mediated interactions between herbivores depends on a number of factors, including the identity of the attacking herbivore (Viswanathan et al. 2005), the identity of the plant (Uesugi et al. 2013; Ali et al. 2014; Rasmann 2014), the identity of the responding herbivore (Soler et al. 2010; Huang et al. 2014), and the sequence of arrival (Erb et al. 2011b; Wang et al. 2014). Given the substantial spatial and temporal variation in herbivore communities in nature (Huntly 1991), the question arises whether plant‐mediated interactions are predictable for the involved players (Soler et al. 2013) or whether they are largely stochastic (van Dam and Heil 2011). Predictability can favor adaptive evolution, and in the case of plant‐mediated interactions, stable patterns may prompt natural enemies to use cues to select good hosts (de Rijk et al. 2013) and plants to adjust to changing herbivore preference and attack patterns (Anderson et al. 2011). Therefore, if we are to interpret ecological observations in relation to plant‐mediated interactions from an evolutionary point of view, knowledge about the relative predictability of the underlying herbivore–plant–herbivore interplay is important. The same holds true for the integration of plant‐mediated interactions into pest forecasting models and integrated pest management strategies.

Several studies have addressed specificity in plant‐mediated interactions by varying one or several dimensions of the interaction (Viswanathan et al. 2005; Soler et al. 2007; Ali et al. 2014). However, comparatively few studies have systematically varied all three dimensions (i.e., the attacking herbivore, the responding plant, and the responding herbivore). Uesugi et al. (2013) investigated plant‐mediated interactions between three specialist herbivores in three *Solidago altissima* (L.) genotypes and found significant differences in elicitation and response patterns, but no clear influence of plant genotype on the herbivore responses. Huang et al. (2014) documented that herbivore identity determines the direction of plant‐mediated interactions in tallow trees (*Triadica sebifera* L.) in the laboratory and the field, with effects ranging from inhibition to facilitation. A subset of the patterns differed between tree populations from different origins (Huang et al. 2014), suggesting an influence of plant genotype. Currently, the small numbers of different species and genotypes used in most studies make it difficult to assess the presence of general patterns in any given plant system.

**Figure 1:**
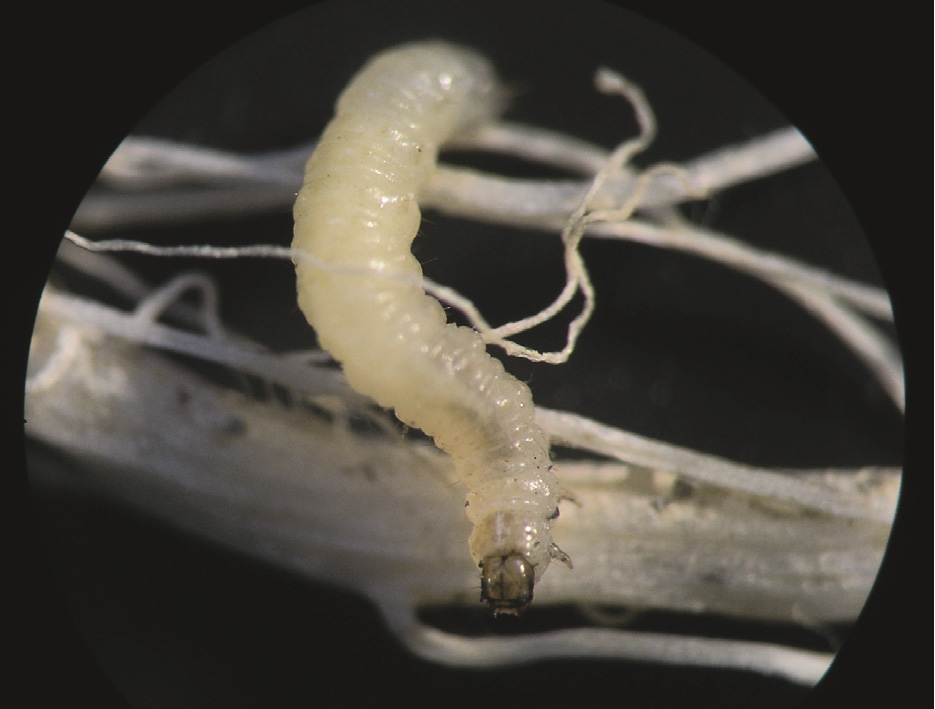
A first instar western corn rootworm larva (*Diabrotica virgifera virgifera*) attacking a maize root. Picture credit: Christelle A.M. Robert.

Here, we investigated the specificity of plant‐mediated interactions between leaf‐chewing and root‐feeding herbivores in maize (*Zea mays* subsp. *mays* L.). Interactions between above‐ and belowground herbivores are well suited to assess plant‐mediated interactions, as herbivores are spatially separated and therefore do not physically interact. Our previous work has shown that root attack by the larvae of the chrysomelid beetle *Diabrotica virgifera virgifera* (LeConte) (Fig. 1) reduces leaf damage in the field and increases leaf resistance against the leaf‐feeding larvae of the noctuid moth *Spodoptera littoralis* (Boisduval) and the necrotrophic pathogen *Setosphaeria turcica* (Luttr.) (Erb et al. 2009, 2011a). In the opposite direction, *Spodoptera frugiperda* (Smith) and *S. littoralis* attack reduces the performance of *D. v. virgifera* in the laboratory and the field (Gill et al. 2011), especially if the leaf feeder attacks the plant before the root feeder (Erb et al. 2011b). Although maize is a domesticated plant, much of the genetic diversity of its wild ancestor, teosinte, has been preserved (Hufford et al. 2012). Furthermore, our previous work shows that the interaction between *Spodoptera* spp. and *D. v. virgifera* is similar in cultivated maize and teosinte (Erb et al. 2011b). Here, we addressed the question whether the negative effect of leaf herbivore attack on root feeders in maize is a general pattern or whether it only occurs for the previously investigated combinations of herbivores. We measured root herbivore preference, which is a good predictor of root damage, biomass consumption, and survival of *D. v. virgifera* (Erb et al. 2011b; Robert et al. 2012b). By testing the effect of herbivore‐specific induction using oral secretions of six different leaf feeders on the response of three different maize‐associated herbivores in nine different maize genotypes, we uncover a strongly conserved interaction pattern which suggests that the outcome of plant‐mediated interactions between chewing herbivores in maize is highly predictable.

## Materials and Methods

### Plants and insects

The maize hybrid Delprim was obtained from Delley DSP (Delley, Switzerland). Parental inbred lines of the maize nested association mapping (NAM) population, including the lines B73, CML103, HP301, Ki11, Mo18w, NC358, P39GB, and Tx303, were obtained from the Maize Genetics Cooperation Stock Center (COOP). Plants were grown in a bottom‐pierced plastic pots (diameter: 4 cm; depth: 11 cm) filled with sand (Hagebaumarkt Leipzig GmbH, Leipzig, Germany) and 1 cm of potting soil (Tonsubstrat, Geeste, Germany) on top in a greenhouse (26 ± 2°C, 14‐h photoperiod, 55% relative humidity). Plants were irrigated daily with tap water and fertilized twice a week with 0.1% Ferty 3 (Planta Düngemittel, EUFLOR GmbH, München, Germany). Twelve‐ to fourteen‐day‐old plants with four fully developed leaves (growth stage V3) were used for all experiments. Eggs of *Diabrotica balteata* (LeConte) and *Diabrotica v. virgifera* were obtained from Syngenta (Stein, Switzerland) and the USDA‐ARS (Brookings, SD, USA). Eggs of *D. undercimpunctata howardii* (L.) were provided by Crop Characteristics Inc. (Farmington, MN, USA). All species were reared on fresh maize seedlings (hybrid Biotop, Maisadour Semences, Mont‐de‐Marsan, France) in a climate chamber (25 ± 2°C, 14‐h photoperiod, 60% relative humidity) until use. *S. littoralis*,* Helicoverpa armigera* (Hübner), *Lymantria dispar* (L.)*, Mamestra brassicae* (L.), *S. frugiperda,* and *Manduca sexta* (L.) larvae were obtained from in‐house rearings at the Max Planck Institute for Chemical Ecology (Jena, Germany). Third to fourth instar larvae were fed on maize (Delprim) for at least 24 h before collecting their oral secretions (OSs), with the exception of the specialist *M. sexta*, which refused to feed on maize plants and was therefore kept on wild tobacco (*Nicotiana attenuata,* Torr. ex Watson) until OS collection. Oral secretions were collected as described (Erb et al. 2009).

### Plant treatments

To induce plants in a controlled manner, we mimicked leaf herbivore attack by wounding the undersides of maize leaves by scratching a surface of 2 cm on each side of the midrib with a razor blade. Five microliters of oral secretions (W+OS) or H_2_O (W) was then applied to the wounds with a micropipette. W+OS treatment results in plant responses that are similar to leaf herbivore attack in maize (Erb et al. 2009). Unless otherwise specified, we first elicited the lowest leaf (L0), followed by the next upper leaf every 12 h. In this way, all the leaves (L0–L3) were elicited after 48 h. The wounding sequence corresponds to the sequential damage pattern of *S. littoralis* on maize plants (Köhler et al. 2015). To test whether the behavioral response of the root feeders depends on the site of elicitation, we treated only one leaf per plant four times over 48 h by wounding the leaf surface from the tip to the base in a separate experiment (see below). For all experiments, plants without leaf elicitation were included as controls. Root herbivore preference experiments were carried out 48 h after the beginning of the leaf elicitation treatments.

### Herbivore preference setup

The feeding preference of root herbivores was determined in the greenhouse using a Petri dish setup as described (Robert et al. 2012b). This setup allows for the rapid screening of root herbivore preference and results in similar patterns compared to soil‐based systems (Robert et al. 2012b; Erb et al. 2015). Whole plants were removed from the pots and their roots were carefully washed with distilled water. Roots of two plants with different leaf treatments (see below) were laid onto a moist filter paper embedded in a Petri dish. Six second instar *Diabrotica* larvae were then introduced into the Petri dish, which was rapidly closed again and sealed with aluminum foil. The stems were laid in a cavity on the side of the dish, allowing the aboveground parts to be laid freely on the greenhouse tables. The number of larvae on the root systems of the different plants was recorded 0.5, 1.5, 3, and 4 h after introduction. This system delivers similar results to soil‐based preference setups (Robert et al. 2012b).

### Herbivore preference tests

Using the setup described above, we carried out a series of preference tests in a partial factorial design. We first defined a generic interaction system consisting of (1) the maize hybrid Delprim, for which herbivore‐induced defense responses are well characterized (Rasmann and Turlings 2007; Ton et al. 2007; Erb et al. 2009), (2) the leaf‐feeding larvae of *S. littoralis*, which feed on a variety of host plants, including maize (Salama et al. 1971), and (3) the root‐feeding larvae of *D. balteata*, which have an equally diverse host range, including maize (Pitre and Kantack 1962). Starting from this system, we then varied the identity and elicitation pattern of the inducing herbivore, the identity of the responding herbivore, and the genetic background of the mediating plant. First, we tested herbivore specificity by evaluating the preference of *D. balteata* between control and wounded plants with (W+OS) and without (W) *S. littoralis* OS (var. Delprim, *n* = 18 per induction type). Next, we tested the influence of leaf attack position by evaluating the preference of *D. balteata* between control and *S. littoralis* W+OS‐induced maize plants using seedlings (var. Delprim) that were treated on Leaf 0 (L0, the first emerging leaf, sometimes also referred to as cotyledon), L1, L2, or L3 (*n* = 18 for each site of induction). To test the importance of the identity of the inducing herbivore, we tested the preference of *D. balteata* between control and W+OS‐induced plants (var. Delprim), including plants that were treated with the OS from *H. armigera*,* L. dispar, M. brassicae*,* S. frugiperda*, and *M. sexta* (*n* = 18 for each species). *H. armigera* and *S. frugiperda* are generalist herbivores that feed on maize in the field. *L. dispar* is a generalist feeding on a number of tree species. *M. brassicae* is a generalist with a preference for cabbage plants, and *M. sexta* is specialized on Solanaceae. To test the importance of the identity of the responding herbivore, we evaluated the preference of *D. v. virgifera*,* D. balteata*, and *D. undecimpunctata* when given a choice between control and *S. littoralis* W+OS‐induced maize plants (var. Delprim, *n* = 13). Finally, we tested the influence of plant genotype by evaluating the preference of *D. balteata* between control and *S. littoralis* W+OS‐induced maize seedlings with different genetic backgrounds. Several parental lines of the maize nested association mapping (NAM) population, which were selected to cover a large portion of maize genetic diversity (McMullen et al. 2009), were used, including the inbred lines B73 (*n* = 17), CML103 (*n* = 18), HP301 (*n* = 12), Ki11 (*n* = 18), Mo17 (*n* = 18), NC358 (*n* = 18), P39 (*n* = 16), and Tx303 (*n* = 18). A detailed characterization of these lines can be found elsewhere (McMullen et al. 2009). Note that while these parental lines are an ideal resource to screen the overall impact of maize genetic diversity on a given trait, they do not allow for a more detailed association between genetic and phenotypic traits due to genetic linkage disequilibria.

### Data analysis

To analyze root herbivore preference, we calculated choice proportions for each independent replicate by dividing the average number of total feeding larvae by the average number of larvae feeding on control roots. The proportions of larvae choosing the control sides were then compared to the null hypothesis (equal preference for both sides, resulting in an average proportion of 0.5) using one‐sample *t*‐tests in R. Within experiments, levels of significance were adjusted for multiple testing by applying the false discovery rate (FDR) method described by Benjamini and Hochberg (1995) using an Excel worksheet available under the Creative Commons Attribution‐NonCommercial‐ShareAlike 3.0 Unported License (Manuel Weinkauf, Zentrum für Marine Umweltwissenschaften, Germany). Within experiments, different choice ratios were compared through analysis of variance (ANOVA) followed by Tukey contrasts in R. Assumptions for ANOVAs were verified using Shapiro–Wilk and Levene's tests in SigmaPlot 12.0 (Systat Software, Erkrath, Germany).

## Results

**Figure 2:**
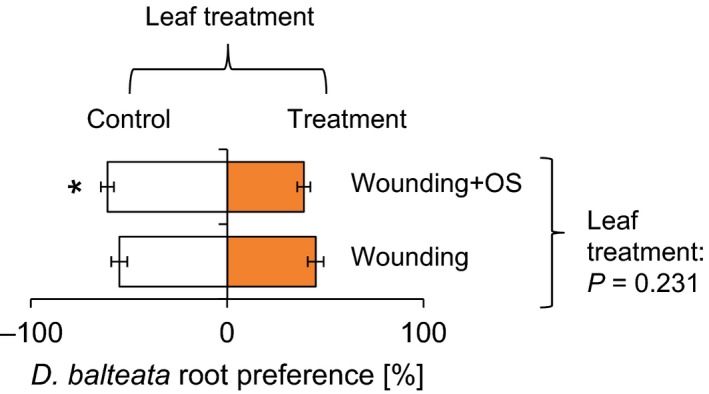
*Diabrotica balteata* specifically avoids roots of leaf‐infested plants. Average root preferences (±SE) of dual‐choice experiments are shown. Leaves were induced by wounding or wounding and application of *S. littoralis* oral secretions. Stars indicate a significant preference for control plants (FDR‐corrected *q* < 0.025). The *P*‐value of an analysis of variance comparing the different choice situations is shown on the right.

In a first experiment, we compared the response of *D. balteata* to leaf elicitation by wounding and wounding combined with the application of *S. littoralis* oral secretions. The oral secretions of *S. littoralis* contain elicitors that induce a plant response similar to real herbivore attack (Erb et al. 2009), enabling us to evaluate whether *D. balteata* responds to leaf damage or, more specifically, to the plant's reaction to exposure to herbivore‐associated molecular patterns (HAMPs). When given a choice between control and wounded plants, *D. balteata* showed no clear preference (Fig. 2, FDR‐corrected *q* = 0.025, *t* = −1.24, *P* = 0.2305): Larvae were found to feed on the roots of both plants with a nearly equal frequency. By contrast, *D. balteata* larvae avoided plants that were induced by wounding and application of oral secretions and showed a significant preference for control plants (*t* = −3.38, *P* = 0.003). No significant difference in preference was found when comparing the choice ratios of the two treatments (*F* = 1.39, *P* = 0.246).

**Figure 3:**
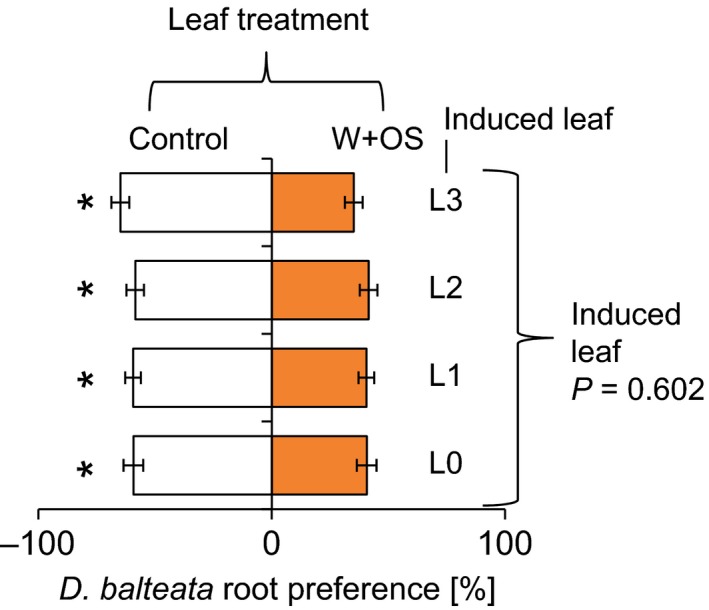
*Diabrotica balteata* avoids leaf‐infested plants independently of the site of attack. Average root preferences (±SE) of dual‐choice experiments are shown. Individual leaves were induced by wounding and application of *S. littoralis* oral secretions. Stars indicate a significant preference for control plants (FDR‐corrected *q* < 0.05). The *P*‐value of an analysis of variance comparing the different choice situations is shown on the right.

Different herbivores show different aboveground feeding patterns (Köhler et al. 2015). To understand whether the site of induction is important for the elicitation of the root herbivore preference pattern, we elicited individual leaves of maize plants by wounding and application of *S. littoralis* oral secretions and measured *D. balteata* preference (Fig. 3, FDR‐corrected *q* = 0.05). Irrespective of the elicited leaf, *D. balteata* avoided the roots of leaf‐induced plants (L0: *t* = 2.26, *P* = 0.037; L1: *t* = 2.9, *P* = 0.011; L2: *t* = 2.3, *P* = 0.033; L3: *t* = 4.00, *P* < 0.001). No significant differences were found between the choice ratios of the different elicitation positions (*F* = 0.63, *P* = 0.602).

**Figure 4:**
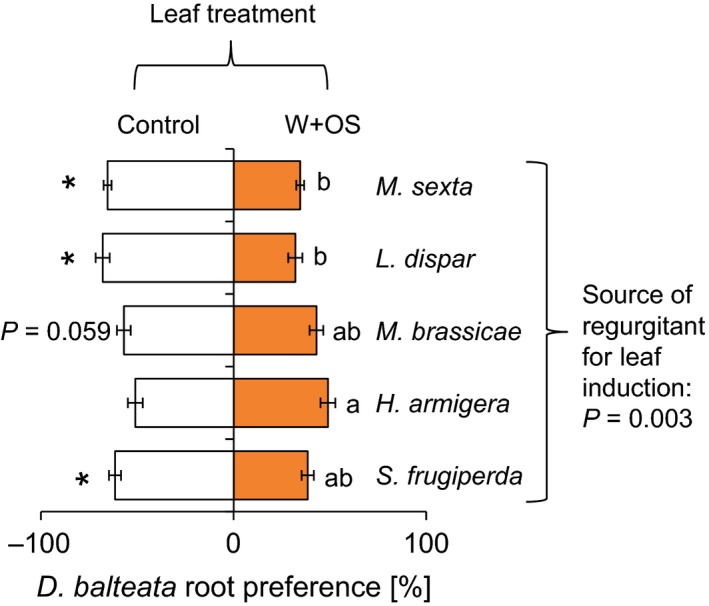
Leaf herbivore identity determines *D. balteata* root preference. Average root preferences (±SE) of dual‐choice experiments are shown. Plants were induced by wounding and application of oral secretions of different leaf‐chewing herbivores. Stars indicate a significant preference for control plants (FDR‐corrected *q* < 0.03). Raw *P*‐values for *P* ≤ 0.1 are depicted. The *P*‐value of an analysis of variance comparing the different choice situations is shown on the right.

To assess whether HAMPs from other leaf‐chewing herbivores elicit different preference responses in *D. balteata*, we treated wounded plants with oral secretions from five additional herbivores, ranging from the common maize feeder *S. frugiperda* to the solanaceous specialist *M. sexta* (Fig. 4, FDR‐corrected *q* = 0.03). *D. balteata* larvae showed a significant preference for control roots when offered plants that were elicited by *S. frugiperda* (*t* = −3.77, *P* = 0.001), *M. sexta* (*t* = −7.8, *P* < 0.001), and *L. dispar* (*t* = −5.1, *P* < 0.001). They also tended to prefer controls of *M. brassicae*‐induced plants (*t* = −2.0, *P* = 0.059). By contrast, no significant avoidance response toward *H. armigera*‐induced plants was found (*t* = −0.3, *P* = 0.784). Analysis of variance revealed a significant treatment effect (*F* = 4.4, *P* = 0.003), and Tukey contrasts showed a pairwise difference between the choice rates in the *H. armigera* and *M. sexta* (*P* = 0.02) as well as *L. dispar* treatments (*P* = 0.003).

**Figure 5:**
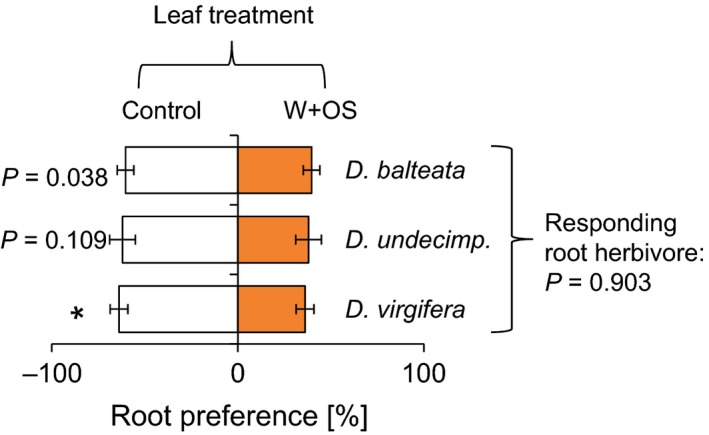
Root herbivore preference patterns do not differ between Diabrotica species. Average root preferences (±SE) of dual‐choice experiments are shown. Plants were induced by wounding and application of *S. littoralis* oral secretions (Star indicates an FDR‐corrected *q* < 0.016). Raw *P*‐values for *P* ≤ 0.1 are depicted. The *P*‐value of an analysis of variance comparing the different choice situations is shown on the right.

Maize is attacked by several root‐feeding insects, including the specialist *D. v. virgifera* and the polyphagous *D. balteata* and *D. undecimpunctata* (O'Day 1998). To assess whether these species respond differently to leaf elicitation, we profiled their behavior in a choice setup including root systems from control and *S. littoralis* W+OS‐elicited plants (Fig. 5, FDR‐corrected *q* = 0.016). *D. v. virgifera* significantly preferred control over leaf‐infested plants (*D.v.v*:* t* = −2.9, *P* = 0.012). *D. balteata* and *D. undecimpunctata* showed trends in the same direction (*D.b*:* t* = −2.3, *P* = 0.038; *D.u*:* t* = −1.7, *P* = 0.109). No significant difference was found between the choice ratios of the different herbivores (*F* = 0.9, *P* = 0.903).

**Figure 6:**
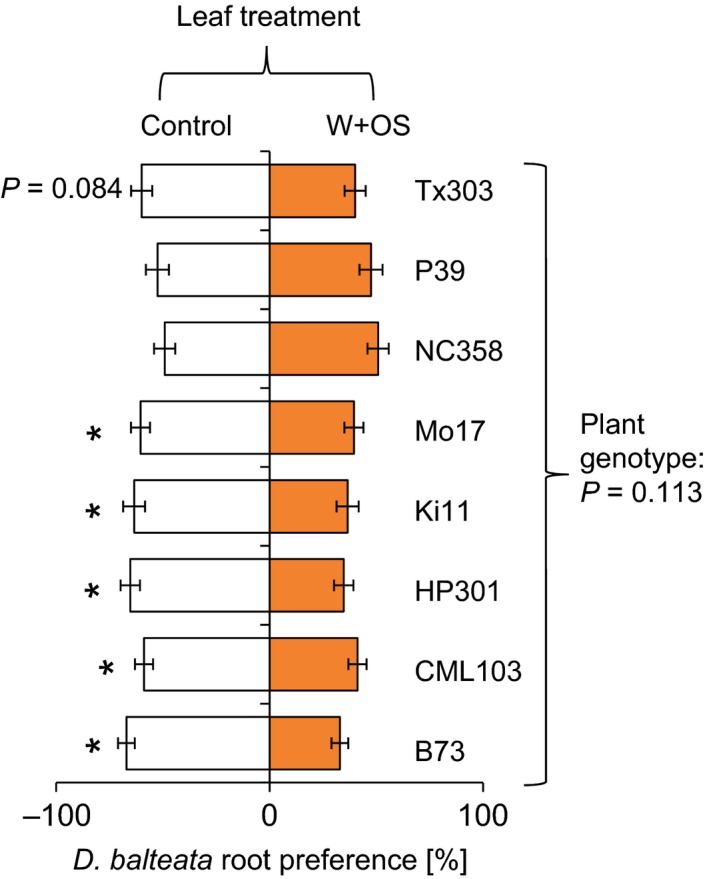
The plant genetic background determines *D. balteata* root preference. Average root preferences (±SE) of dual‐choice experiments are shown. Inbred lines with different genetic backgrounds were induced by wounding and application of *S. littoralis* oral secretions. Stars indicate a significant preference for control plants (FDR‐corrected *q* < 0.03). Raw *P*‐values for *P* ≤ 0.1 are depicted. The *P*‐value of an analysis of variance comparing the different choice situations is shown on the right.

To understand the influence of the plant genetic background on the leaf‐induced root herbivore preference pattern, we used eight parental lines of the nested association mapping population, including germplasm from North America, Mexico, and the tropics (McMullen et al. 2009). In five genotypes, *D. balteata* preferred control over leaf‐induced plants (Fig. 6; FDR‐corrected *q* = 0.031, *P* < 0.031). In one line, Tx303, the larvae showed a tendency for controls (*t* = −1.8, *P* = 0.084). In two lines, P39 and NC358, no significant preference pattern was observed (P39: *t* = −0.48, *P* = 0.636; NC358: *t* = 0.17, *P* = 0.866). The overall effect of plant genotype on choice ratios was not significant (*F* = 1.7, *P* = 0.113).

## Discussion

**Figure 7:**
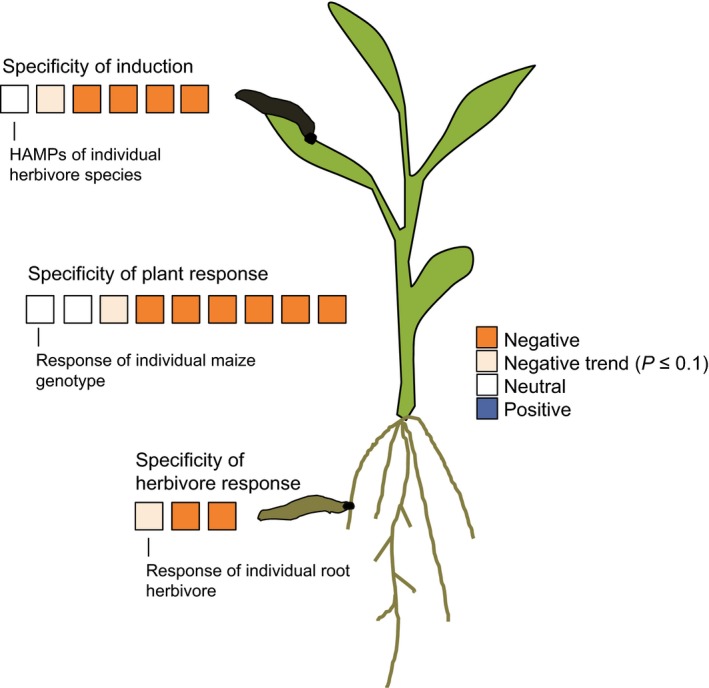
A conserved pattern of plant‐mediated interactions between herbivores in maize. The figure summarizes the direction of the effects of leaf induction by different herbivores on the responses of different root herbivores in the different plant genetic backgrounds that were tested in this study. Note that only neutral to negative effects were observed; no combination resulted in an increase of attractiveness of the roots for the root herbivores.

The present study reveals that plant‐mediated interactions between leaf‐ and root‐feeding herbivores are conserved across different inducing and responding herbivore species and plant genotypes (Fig. 7). For the large majority of tested leaf herbivores, plant genotypes, and root herbivores, the outcome of the interaction was the same: Leaf elicitation resulted in a reduction in root attractiveness and a preference of the root feeders for control plants. This result is in line with earlier studies showing that leaf attack by *Spodoptera* spp. reduces the number of *D. v. virgifera* herbivores feeding on maize roots in the field (Erb et al. 2011b; Gill et al. 2011) and the performance of *D. v. virgifera* on maize and teosinte (Erb et al. 2011b).

In contrast to the general pattern observed here, other studies found substantial differences in the impact of individual herbivore species on plant‐mediated interactions (Poelman et al. 2008; Uesugi et al. 2013; Huang et al. 2014). Two reasons may account for the contrasting results. First, we compared herbivores of the same feeding guild, while many other studies used herbivores with different feeding modes. The feeding guild has been predicted to be an important determinant for the outcome of plant‐mediated interactions (Soler et al. 2013). Second, we standardized leaf damage and specifically compared the influence of the oral secretions of different chewing herbivores. While this approach has its advantages as it specifically measures the response of the plant to different HAMPs (Felton and Tumlinson 2008), it cannot capture the full suite of differences between different attacking herbivores, including bite size, speed, and movement, which may influence plant responses (Bricchi et al. 2010). Johnson et al. (2012) analyzed studies on the outcome of interactions between leaf‐ and root‐feeding herbivores and found that the outcome of the interactions is determined by the sequence of arrival and the type of herbivore. However, the meta‐analysis also documented the substantial variability of outcome parameters that are possible even after separating them by the above parameters (Johnson et al. 2012). We also found that certain combinations of herbivores and genotypes lead to an outcome of the interaction that differs from the norm, which adds to the current notion that most plant‐mediated interactions are, at least to a certain degree, specific. It is also important to note that our experiments only covered a fraction of all possible interaction combinations. In theory, we could have performed 162 different choice experiments with available herbivores and plant genotypes, and it is possible that we may not have captured the full suite of specificity with our partial factorial design.

Understanding whether plant‐mediated interactions lead to stable and predictable outcomes is important to predict their impact on the evolution of associated organisms and to integrate them into pest management strategies. In maize, the entomopathogenic nematode *Heterorhabditis megidis* has been shown to be less attracted to *D. v. virgifera*‐infested roots of plants that are attacked by *S. littoralis* aboveground (Rasmann and Turlings 2007), which may reflect an adaptation to the negative effect of leaf herbivory on maize‐associated root feeders. Furthermore, although maize plants clearly respond systemically to leaf attack, our previous work shows that the systemic response in the roots is fundamentally different from the local leaf response (Erb et al. 2009; Marti et al. 2013). The toxic benzoxazinoid 2‐(2‐hydroxy‐4,7‐dimethoxy‐1,4‐benzoxazin‐3‐one)‐beta‐D‐glucopyranose (HDMBOA‐Glc) for instance is strongly induced aboveground, but does not respond systemically in the roots (Glauser et al. 2011; Marti et al. 2013). On the contrary, leaf infestation can even suppress root defenses, including the release of benzoxazinoids and terpenoids (Rasmann and Turlings 2007; Neal et al. 2012). This pattern of defensive investment may reflect the fact that leaf attack strongly reduces the probability of root infestation, making it unnecessary for the plant to mount costly defense responses belowground other than the systemic changes in phenylpropanoids and ethylene emissions that are likely responsible for the strong avoidance by *Diabrotica* spp. (Robert et al. 2012a; Erb et al., submitted). Finally, *D. v. virgifera* grows significantly less on leaf‐infested maize plants in a no‐choice situation (Erb et al. 2011b). The avoidance response of the root feeders observed here may therefore be an adaptation to the reduced host quality of leaf‐infested plants. Together, the observed responses are compatible with the notion that the stability of the plant‐mediated interaction pattern may have favored adaptive evolution in the associated organisms. As our knowledge about the specificity of plant‐mediated interactions increases, it may eventually be possible to test this hypothesis more comprehensively across different plant systems.

From an applied point of view, our results suggest that it may be possible to adjust treatment thresholds by integrating knowledge about plant‐mediated interactions. As the effect of leaf herbivory on root pests in maize is predominantly negative (Erb et al. 2011b; Gill et al. 2011), it can be expected that the presence of leaf herbivores in the field may make it unnecessary to treat the soil against root feeders. Alternatively, treatment could be restricted to the parts of the field with the lowest density of leaf feeders. Given the substantial cost of *D. v. virgifera* in maize production, it may be warranted to consider refining current pest management strategies accordingly.

## Conclusions

Our study shows that plant‐mediated interactions between chewing herbivores in maize follow a clear pattern: Leaf elicitation reduces the attractiveness of the roots for belowground herbivores. This conserved pattern is associated with potentially adaptive responses of plants, herbivores, and their natural enemies as shown in previous studies, which again is compatible with the notion that stable plant‐mediated interactions can lead to adaptive evolution. In the future, it will be important to extend the current approach involving multiple herbivores and genotypes to other plant herbivore systems to evaluate whether general or stochastic patterns are more prevalent in nature.

## Data Accessibility

To be filled in at a later stage of the review process.

## Conflict of Interest

None declared.
